# Expression of *Vibrio salmonicida* virulence genes and immune response parameters in experimentally challenged Atlantic salmon (*Salmo salar* L.)

**DOI:** 10.3389/fmicb.2013.00401

**Published:** 2013-12-20

**Authors:** Ane M. Bjelland, Aud K. Fauske, Anh Nguyen, Ingvild E. Orlien, Ingrid M. Østgaard, Henning Sørum

**Affiliations:** ^1^Section for Microbiology, Immunology and Parasitology, Department of Food Safety and Infection Biology, Norwegian School of Veterinary ScienceOslo, Norway; ^2^Department of Pharmacy and Biomedical Laboratory Sciences, Faculty of Health Sciences, Oslo and Akershus University College of Applied SciencesOslo, Norway

**Keywords:** *Vibrio salmonicida*, cold-water vibriosis, Atlantic salmon, gene expression studies, virulence factors, innate immune response, two-step RT-qPCR

## Abstract

The Gram-negative bacterium *Vibrio salmonicida* is the causative agent of cold-water vibriosis (CV), a hemorrhagic septicemia that primarily affects farmed Atlantic salmon (*Salmo salar* L.). The mechanisms of disease development, host specificity and adaptation, as well as the immunogenic properties of *V. salmonicida* are largely unknown. Therefore, to gain more knowledge on the pathogenesis of CV, 90 Atlantic salmon parr were injected intraperitoneally with 6 × 10^6^ CFU of *V. salmonicida* LFI1238. Samples from blood and spleen tissue were taken at different time points throughout the challenge for gene expression analysis by two-step reverse transcription (RT) quantitative real-time polymerase chain reaction. Out of a panel of six housekeeping genes, *accD, gapA*, and 16S rDNA were found to be the most suitable references for expression analysis in *Vibrio salmonicida*. The bacterial proliferation during challenge was monitored based on the expression of the 16S rRNA encoding gene. Before day 4, the concentrations of *V. salmonicida* in blood and spleen tissue demonstrated a lag phase. From day 4, the bacterial proliferation was exponential. The expression profiles of eight genes encoding potential virulence factors of *V. salmonicida* were studied. Surprisingly, all tested virulence genes were generally highest expressed in broth cultures compared to the *in vivo* samples. We hypothesize that this general muting of gene expression *in vivo* may be a strategy for *V. salmonicida* to hide from the host immune system. To further investigate this hypothesis, the expression profiles of eight genes encoding innate immune factors were analyzed. The results demonstrated a strong and rapid, but short-lasting innate immune response against *V. salmonicida*. These results suggest that the bacterium possesses mechanisms that inhibit and/or resist the salmon innate immune system until the host becomes exhausted of fighting the on-going and eventually overwhelming infection.

## Introduction

The motile Gram-negative rod *Vibrio salmonicida* is the causative agent of cold-water vibriosis (CV) in farmed Atlantic salmon (*Salmo salar* L.), rainbow trout (*Oncorhynchus mykiss*), and Atlantic cod (*Gadus morhua*) (Egidius et al., 1981, 1986; Holm et al., 1985; Jørgensen, 1987). The disease occurs mainly in late autumn to early spring and is a generalized septicemia characterized by anemia and extended internal and external hemorrhages (Holm et al., 1985; Poppe et al., 1985; Egidius et al., 1986). Although *V. salmonicida* has been known in Norwegian aquaculture for more than 25 years, only a few studies have so far identified components with possible roles in virulence. These include a surface antigen VS-P1, temperature-sensitive iron sequestration, possible production of hydrogen peroxide, quorum sensing and motility (Hjelmeland et al., 1988; Fidopiastis et al., 1999; Colquhoun and Sørum, 2001; Karlsen et al., 2008; Bjelland et al., 2012a,b). In addition, genomic analysis has identified three putative hemolysins, proteases and several protein secretion systems (Hjerde et al., 2008). The bacterium has, however, been described to be a poor producer of proteases and hemolysins, and a capacity of producing extracellular toxins has never been identified (Holm et al., 1985; Hjelmeland et al., 1988; Toranzo and Barja, 1993; Bjelland et al., 2012b).

Several studies have tried to uncover the pathogenicity of *V. salmonicida* (Totland et al., 1987; Espelid et al., 1988; Bøgwald et al., 1990; Evensen et al., 1991; Brattgjerd and Evensen, 1996). After challenge, *V. salmonicida* has been described to rapidly establish a bacteremia. Before the fish shows clinical signs of disease, bacterial cells have only been detected in the blood stream (Totland et al., 1987; Bjelland et al., 2012a). This latency period can persist up to 5–10 days in artificially infected fish. During this time it has been suggested that *V. salmonicida* uses the blood stream to proliferate and ensure a successful infection (Bjelland et al., 2012a). The first targets of *V. salmonicida* are reported to be the endothelial cells of capillaries and leukocytes of the blood. In the later stages of infection endothelial cells are completely disintegrated and actively proliferating bacteria can be detected in the extravascular space and in the surrounding tissue (Totland et al., 1987).

Little is known about the immune response toward *V. salmonicida* infections in salmonid fish. Previous investigations have mainly targeted the humoral immune response in a variety of vaccination studies. The dominant antigen VS-P1 has been described to specifically stimulate B lymphocytes and antibody production (Espelid et al., 1987; Espelid and Jørgensen, 1992). Although a strong humoral immune response against *V. salmonicida* is demonstrated and suggested to be of protective nature, a poor correlation between protection and antibody production in immune responses against *V. salmonicida* has been obtained (Lillehaug et al., 1993; Eggset et al., 1997). Good efficacy of fish vaccines in the absence of detectable antibodies has been postulated to be T-cell mediated (Eggset et al., 1997).

The threat of *V. salmonicida* to the fish farming industry has been mitigated by vaccination. However, a significant increase of CV outbreaks has recently been reported suggesting a reemerging pathogen (Johansen, 2013). If the pathogen were to reemerge, our lack of knowledge on the virulence mechanisms and host immune response would inhibit the development of new counter-measures. Thus, this study was conducted to further elucidate the pathogenesis of *V. salmonicida*. Due to the lack of identified virulence properties of *V. salmonicida*, we hypothesized that the bacterium may require specific host factors to express important virulence features such as extracellular toxins and adhesins. Therefore, we isolated total RNA from the blood of artificially challenged Atlantic salmon and analyzed the *in vivo* expression of potential virulence genes by two-step RT quantitative real-time (two-step RT-qPCR). To increase our knowledge about the immune responses in Atlantic salmon during CV, the transcription levels of eight innate immune parameters in spleen were evaluated by two-step RT-qPCR. Several hypotheses on the immune response against *V. salmonicida* have previously been formulated. For instance, the bacterium might hide from the host immune system during the latency period of infection, and proliferate. In contrast, in the absence of identified extracellular proteins it has also been suggested that a strong inflammatory response that eventually damage the host's own cells and tissue is responsible for the pathological signs of CV diseased fish (Bjelland et al., 2012a). Thus, this work aimed to verify this hypothesis and to date this is the first report on the innate immune response against a *V. salmonicida* infection.

## Materials and methods

### Fish and holding conditions

Ninety unvaccinated Atlantic salmon parr of approximately 50 g and 8 months old were obtained from Sørsmolt AS (Sannidal, Norway). The fish were transported to the aquarium at the Norwegian School of Veterinary Science (Oslo, Norway) in a tank containing 800 l of oxygenized freshwater with a dissolved oxygen concentration between 8 and 13 ppm. The salmon were kept in a 600 l tank supplied with well aerated freshwater purified on a carbon filter medium (Pentair Water, Minneapolis, MN, USA) at a temperature of ≈7°C and with an oxygen saturation between 10 and 11 ppm. The fish were fed *ad libitum* and acclimatized to the experimental conditions 2 weeks prior to challenge.

### Bacteria and challenge procedure

*V. salmonicida* wild type strains LFI1238 genome sequenced strain were taken from freeze stocks at −70°C and cultivated in Luria Bertani broth with 1% NaCl (LB1) with agitation at 200 rpm at 8°C for 2 days. To verify and prepare virulence of the bacterial strain used in challenge, three Atlantic salmon parr were anaesthetised in a water bath containing 0.0035% benzocaine (*Benzoak*® VET, Euro-Pharma, Chemainus, Canada) and injected intraperitoneally (i.p.) with 0.1 ml of the bacterial culture. The fish showed severe symptoms of CV 4 days post infection and were euthanized and post mortem examined. The bacterium was recovered by taking bacteriological samples from the head kidney using a sterile metal loop, plated on Blood Agar Base No. 2 (Oxoid, Cambridge, UK) supplemented with 5% ox blood and 2.5% NaCl (BA2.5) and incubated at 8°C for 4 days. *V. salmonicida* LFI1238 strain directly isolated from the diseased fish was then cultivated for the gene expression experiment as described above. The challenge experiment was carried out with a lethal dose of *V. salmonicida* that was expected to kill approximately 80–90% of the fish. Under these conditions, the fish usually develop symptoms of disease after 4–5 days followed by mortalities from day 5–6 post infection (Nordmo et al., 1997; Nelson et al., 2007; Bjelland et al., 2012a,b). Ninety Atlantic salmon parr were anaesthetised as described above and divided in one test group of 70 fish and one control group of 20 fish. The fish fins were differentially clipped to distinguish between groups. After sedation, the fish were injected i.p. with 0.1 ml of cultures of strain LFI1238 grown on LB1 with absorbance at 600 nm (A600) of 0.3 representing 6 × 10^6^ CFU. The final bacterial suspension was controlled by colony counting following serial dilution in LB1, plating of aliquots of 100 μl in duplicate on BA2.5 and incubation at 8°C for 4 days. The control fish were injected i.p. with 0.1 ml phosphate buffered saline (PBS). The challenge experiment was approved by The Norwegian Animal Research Authority (approval no. ID4877).

### Sampling

Before challenge, 200 μl of the final bacterial culture was transferred to 400 μl of RNAlater (Ambion, Applied Biosystems, Foster City, CA, USA) in triplicate, vortexed and incubated for 5 min at room temperature followed by pelleting at 8600 rpm for 10 min using a Himac CT15RE tabletop centrifuge (Hitachi Koki Co., Ltd., Tokyo, Japan). The bacterial pellet was then stored at −20°C until RNA isolation. At different time points starting from 2 h to day 6 after challenge, five fish were removed from the test tank and euthanized in a water bath containing 0.01% benzocaine. Blood samples were taken from the caudal vein using vacutainer blood collection tubes with EDTA anticoagulants. The procedure was followed by gently inverting the collection tubes several times to allow for anticoagulation. A volume of 200 μl anticoagulated blood was then thoroughly mixed with 1 ml of RNAlater in a microfuge tube, incubated in room temperature for 1 h before pelleting (1 min, 13.000 rpm min) using a Himac CT15RE and stored at −20°C until RNA isolation. In addition to blood sampling, the spleen was dissected and transferred to 1 ml RNAlater. The tissue samples were incubated for 24 h at 4°C before stored at −20°C until RNA extraction. Further, bacteriological samples from the blood and head kidney were plated on BA2.5 and incubated at 8°C for 4 days. The presence of *V. salmonicida* and other bacterial species from each sample were graded based on the four-quadrant semi-quantitative scoring method (Cappuccino and Sherman, 2007). The first quadrant of the plate was streaked using a sterile metal loop and each successive quadrant was streaked using a new bacteriologic plastic loop in order to dilute the number of bacteria in each quadrant. Quantification was expressed as 1+, 2+, 3+, or 4+ based on the number of quadrants that demonstrated bacterial growth. Growth of *V. salmonicida* limited to quadrant 1 was categorized as 1+ (sparse amounts), bacterial growth limited to quadrants 1 and 2 was categorized as 2+ (moderate amounts), bacterial growth limited to quadrants 1, 2, and 3 was categorized as 3+ (rich amounts), and bacterial growth that extended to all four quadrants was categorized as 4+ (very rich amounts).

Identical sampling procedure was performed to 10 control fish; five fish before start and five fish at the end of the challenge. No moribund fish were sampled for gene expression experiments.

### RNA extraction

Total RNA from bacterial pellets was extracted using the RNAeasy Mini Kit (Qiagen, Hilden, Germany) according to the manufacturer's instructions (Protocol 4 and 7) including an on-column DNA wipeout treatment (Appendix B1-4). To extract total RNA from blood, samples were incubated in 1 ml TRIzol reagent (Invitrogen, Carlsbad, CA, USA) for 10 min with regularly vortexing. Spleen samples were transferred to tubes with 5 mm stainless steel beads (Qiagen). Then, 1 ml TRIzol reagent was added followed by homogenization using TissueLyser II (Qiagen) at 25 Hz for 4 min. Further, 200 μl chloroform was added to the blood and spleen samples. The samples were shaken by hands for 15 s, incubated at room temperature for 5 min and centrifuged at 11.400 rpm for 15 min at 4°C min using a Himac CT15RE. A volume of 400–500 μl of the aqueous phase was then mixed 1:1 with 70% ethanol, transferred to an RNAeasy® spin column (Qiagen). Total RNA was then isolated according to the manufacturer's protocol for extraction of total RNA from animal tissues using an RNAeasy® mini kit. The RNA was eluted in 30 μl DEPC-treated water (Invitrogen) and stored at −70°C until reverse transcription (RT). Gel electrophoresis with 1% agarose gel was used to confirm that isolated RNA was intact while the concentration and purity of the RNA extracts were analyzed by measuring the absorbances at 260 (A260) and 280 nm (A280) using a NanoDrop™ ND-1000 spectrophotometer (Thermo Scientific, Waltham, MA, USA). Only total RNA samples of high quality with A260/A280 ratios between 1.9 and 2.2 and with tight bands of 18S/28S ribosomal RNA (rRNA) were used for RT.

### Two-step reverse transcription quantitative real-time polymerase chain reaction (two-step RT-qPCR)

RT was conducted with QuantiTect® RT kit (Qiagen) according to manufacturer's instructions for the synthesis of complementary DNA (cDNA) and included a DNase wipeout treatment. Amounts of 1 μg of RNA were used in each RT reaction conducted in a BioRad T100 (Bio-Rad, Hercules, CA, USA). In addition, to confirm the absence of any contamination with genomic DNA (gDNA) contamination, one RNA sample per round of extraction was randomly chosen and not treated with reverse transcriptase. The cDNA samples were diluted in 180 μl of DEPC-treated water and stored at −70°C until quantitative real-time PCR (qPCR). The qPCR was performed using EXPRESS SYBR® GreenER™ qPCR Supermixes (Invitrogen) according to the manufacturer's instructions. The reactions were performed in triplicate with a Stratagene Mx3005P (Stratagene, La Jolla, CA, USA) detection system at the following conditions: 50°C for 2 min, 95°C for 2 min, 40 cycles of 95°C for 15 s and 60°C for 60 s, followed by 95°C for 1 min, 55°C for 30 s, 95°C for 30 s (dissociation curve). Each plate included a no-template control for non-specific amplification that comprised DEPC-treated water instead of cDNA. Each primer pair was shown to have no primer dimer product in the no-template control and a single peak in the dissociation curve. Randomly chosen samples that were not treated with reverse transcriptase were tested to confirm any absence of contamination by gDNA. Data were captured using the Stratagene MxPro Mx3005P QPCR software. The potential virulence genes surveyed in this work were chosen to elucidate the role of extracellular toxins, secretion systems and adhesion factors in the pathogenesis of *V. salmonicida*. In addition, a panel of eight key innate immune genes was chosen based on previous reports to investigate the host's early response to a CV infection. Primer sequences targeting for the genes coding for 16S rRNA, AccD, FstZ, RpoD, LitR, EFN1β, β-actin, 18S rRNA, TLR5S, TNFα, IL-1β, IL-6, IL-8, IL-12, IFNα, and C3 were obtained from previous publications (Olsvik et al., 2005; Løvoll et al.,^2007^; Hynes et al., 2011; Bjelland et al., 2012b). Other primers were designed from sequences found in GenBank using the Primer3 online program (Table 1). One sequence was used for each primer designed. Accession numbers (NCBI) for the sequences are as follows: *polA*, gi: 6989219; *gapA*, gi: 6987419; *vah2*, gi: 6988754; *vah5*, gi: 6986473; *hlyIII*, gi: 6986281; *tadA*, gi: 6962598; *vasA*, gi: 6989384; *tolC*, gi: 6961545; *epsD*, gi: 6988447. All primers were purchased from Invitrogen with standard desalting.

**Table 1:** **Primers used in this study**.

**Protein**	**Primer**	**Primer sequence (5′-3′)**	**Ref.**
16S ribosomal RNA	16S rDNA-F	CTTGACGTTAGCGACAGAAGAA	^**^
	16S rDNA-R	CGCTTTACGCCCAGTAATTC	
Acetyl-CoA carboxylase subunit beta	accD-F	TTGCTGGTCGTCGTGTTATT	^**^
	accD-R	TTTAGCCATCAAACCACCAA	
Cell division protein FtsZ	ftsZ-F	CGGATGTTCGTACGGTAATG	^**^
	ftsZ-R	CAAGCAATGGGCTTGAGATA	
RNA polymerase sigma factor RpoD	rpoD-F	AAGCCGAAGAAATTCGCTTA	^**^
	rpoD-R	GCAAGATCTGATTCGCTCAA	
Glyceraldehydes-3-phosphate dehydrogenase	gapA-F	TTTGTTTTCCGTGCATCTGT	^*^
	gapA-R	GTTGAAACGACCGTGAGTTG	
DNA polymerase I	polA-F	TTCCTGGCATTGGTGATAAA	^*^
	polA-R	AAACCAAGAGGCGCAATATC	
LitR family transcriptional regulator	litR-F	GCTCAATCCCTTCAGCAAAC	^**^
	litR-R	GTGGTTTGAATGGAGCACCT	
Hemolysin Vah2	vah2-F	GAAATTTCCATGACGCCTTC	^*^
	vah2-R	ACGCCTTTCACAACTGTTCC	
Hemolysin Vah5	vah5-F	CACAAGACAGTCCGGTTCCT	^*^
	vah5-R	ATTGCACCAAAAGCCAATTC	
Hemolysin III	hlyIII-F	AATGGCTGTGATCTGGGCTA	^*^
	hlyIII-R	AACCGATAACCAACCCATGA	
Type VI secretion protein VasA	vasA-F	CAACGCCTGTCGGTAATTCT	^*^
	vasA-R	TGGGGTTCATTGTCAGTTCA	
Putative type I toxin secretion system	tolC-F	CTTTCTGAGCTTGGGTTTCG	^*^
	tolC-R	GCACAATTTCGCTCATCAGA	
Type II secretion pathway protein D	epsD-F	GTGCACCGTAATCACCCTCT	^*^
	epsD-R	CCATGGTGGGCTTAGAAGAA	
Flp pilus assembly protein	tadA-F	ACTTTTCGGCTACCATCGTG	^*^
	tadA-R	CTGCCCTTAGAAGCAATTCG	
Elongation factor 1-beta	EF1β-F	TGCCCCTCCAGGATGTCTAC	^***^
	EF1β-R	CACGGCCCACAGGTACTG	
β-actin	β-actin-F	CCAAAGCCAACAGGGAGAAG	^***^
	β-actin-R	AGGGACAACACTGCCTGGAT	
18S ribosomal RNA	18S rDNA-F	TGTGCCGCTAGAGGTGAAATT	^***^
	18S rDNA-R	CGAACCTCCGACTTTCGTTCT	
Toll-like receptor 5, solid form	TLR5S-F	CCTTGGATCTCCATGGTGTCA	^****^
	TLR5S-R	TGGTTTTGGGTACTTTTCCATTATG	
Tumor necrosis factor alpha	TNFα-F	AATACAACCCCAGTGGATCG	^****^
	TNFα-R	TGTCCTTGTTAGGATGCTTGG	
Interleukin 1-beta	IL-1β-F	GCTGGAGAGTGCTGTGGAAGA	^****^
	IL-1β-R	TGCTTCCCTCCTGCTCGTAG	
Interleukin 6	IL-6-F	ACCAACAGTTTGTGGAGGAGTT	^****^
	IL-6-R	AGCAAAGAGTCTTGGAGAGGTG	
Interleukin 8 (CXCL8)	IL-8-F	ATTGAGACGGAAAGCAGACG	^****^
	IL-8-R	CGCTGACATCCAGACAAATCT	
Interleukin 12	IL-12-F	CTGAATGAGGTGGACTGGTATG	^****^
	IL-12-R	ATCGTCCTGTTCCTCCG	
Interferon alpha	IFNα-F	TGGGAGGAGATATCACAAAGC	^****^
	IFNα-R	TCCCAGGTGACAGATTTCAT	
Complement component 3	C3-F	TCCCTGGTGGTCACCAGTACAC	^*****^
	C3-R	ATGATGGTGGACTGTGTGGATC	

* This study;

** Bjelland et al., 2012b;

*** Olsvik et al., 2005;

**** Hynes et al., 2011;

***** *Løvoll et al., 2007*.

### Data analysis of gene expression

The stability of six house-keeping genes was examined; *accD, fstZ, gapA, polA, rpoD*, and the 16S rRNA encoding gene (hereafter referred to as *16S rDNA*). The *geNorm* VBA applet for Microsoft Excel was used to calculate the gene expression stability measure *M* that is defined as the average pair-wise variation of a particular gene with all other potential reference genes i.e., the least stable gene gets the highest *M*-value (Vandesompele et al., 2002). The threshold cycle (Ct) values were transformed to quantities using the comparative Ct (ΔΔCt) method and the highest relative quantities for each gene was set to 1 in accordance to the *geNorm* manual (Livak and Schmittgen, 2001). From this, a gene expression normalization factor was calculated for each sample based on the geometric mean of the reference genes. Normalized relative gene expression level for each target gene was then calculated by transforming Ct values to quantities using the comparative Ct method and dividing the quantities for each sample by the appropriate normalization factor. Relative fold changes of potential virulence gene transcripts were calculated compared to the bacterial culture before injection into the fish. The relative expression of salmon immune gene transcripts was calculated compared to the control fish which received an injection of PBS. The average of fold change values ± standard error of the mean (s.e.m.) was estimated using the Student's *t*-test and a *p* ≤ 0.05 was considered statistically significant. Statistical analyses and figure construction were performed using the GraphPad Prism 6 software (GraphPad Software, San Diego, CA, USA).

## Results

### Challenge experiment

The bacterial concentrations in blood and head kidney samples of Atlantic salmon parr was by semi-quantitative estimation categorized to 1+ already 2 h after challenge. One day after challenge the concentration were categorized to 2+, and from 4 days post challenge the bacterial growth was categorized to 4+ (data not shown). Mortality was observed at day 6 when the experiment was terminated. *V. salmonicida* was grown in pure culture from the blood and head kidney from all diseased fish. No control fish showed symptoms of infection or mortality and neither *V. salmonicida* nor any other bacterial species were identified after cultivation from this group.

### Evaluation of potential reference genes in two-step RT-qPCR studies of *V. salmonicida*

The *geNorm* VBA applet for Microsoft Excel was used to determine the most stable genes from tested reference genes (Vandesompele et al., 2002). Based on the *M*-values, the stability of the six genes was ranked in the following order: *accD* > *gapA* > *16S rDNA* > *rpoD* > *polA* > *ftsZ*. The geometric mean of the three most stable reference genes was further used to calculate a gene expression normalization factor for each sample. *M*-values for these genes were 0.882 (*accD*), 0.891 (*16S rDNA*), and 1.115 *(gapA*). Previous studies have evaluated the stability of potential reference genes in qPCR studies of Atlantic salmon (Olsvik et al., 2005; Løvoll et al., 2009; Zhang et al., 2011). Based on these results, *EFN1β, β-actin* and the *18S rDNA* was chosen to be used as Atlantic salmon reference genes. The *M*-values in our study were 0.805 (*β-actin*), 0.930 (*EFN1β*), and 1.047 (*18S rDNA*).

### Quantification of *V. salmonicida* growth in tissue samples using two-step RT-qPCR

**Figure 1:**
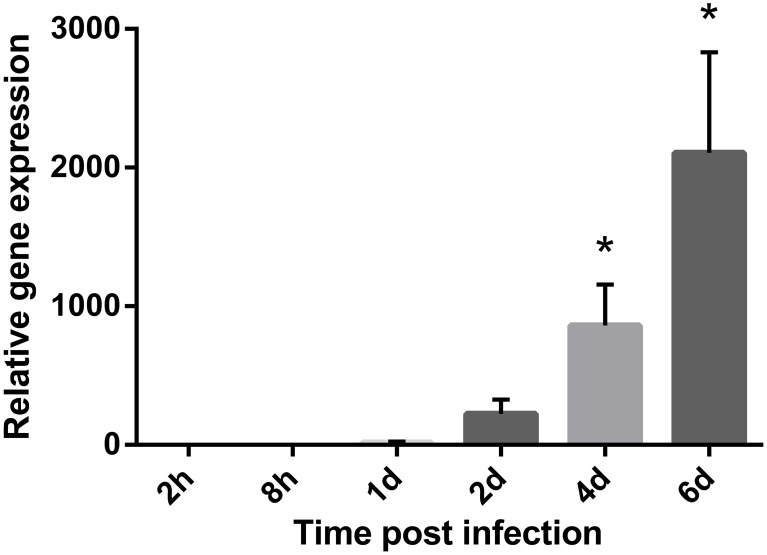
**Bacterial growth during a cold-water vibriosis infection.** Relative expression levels of the 16S rRNA encoding gene (16S rDNA) in the blood of Atlantic salmon parr during an experimental *V. salmonicida* i.p.-infection. Each time point represents the average expression level of the bacterial reference genes in four fish and is reported as fold change averages ± standard error of the mean (s.e.m.) compared to the average expression levels of four control fish 2 h after challenge. ^*^p < 0.05.

The 16S rRNA encoding gene (*16S rDNA*) was by *geNorm* demonstrated to be a stable housekeeping gene. *16S rDNA* was also the highest expressed gene per bacterial cell of all tested housekeeping genes throughout the study. In contrast, the most stable housekeeping gene *accD* demonstrated high Ct values close to the no-template-control (NTC) initially in the experiment. To ensure that the NTC does not contribute to the fluorescence signal of the target sequence, it is recommended that the Ct value of the unknown target gene should have Ct values of 3.3 cycles (a log value) fewer than that of the NTC Ct value (Smith et al., 2006). Thus, the relative expression of *16S rDNA* was used to illustrate the bacterial growth in the fish blood during challenge (Figure 1). The lowest expression of *16S rDNA* was observed at the first sampling point 2 h after challenge (*x* = 1.00 ± 0.93). The relative expression of *16S rDNA* transcripts were therefore calculated compared to this time point. At 8 h post infection the relative expression of *16S rDNA* remained low (*x* = 1.34 ± 0.59). One day after challenge the relative expression of *16S rDNA* increased by 16-fold (*x* = 16.30 ± 6.78). The relative expression continued to increase with time and at 2 and 4 days post infection the relative fold change were 223 (*x* = 223.15 ± 103.36) and 861 (*x* = 860.77 ± 294.82) higher, respectively. Overall, the highest increase in relative expression was seen on day 6 after challenge and was at this time point 2107-fold higher compared to the initial sampling point (*x* = 2107.36 ± 724.90). Statistical significant results were observed on day 4 (*p* = 0.03) and 6 (*p* = 0.03). No transcription products of *16S rDNA* were identified in the control fish.

### Expression of potential *V. salmonicida* virulence genes during a cold-water vibriosis infection

**Figure 2:**
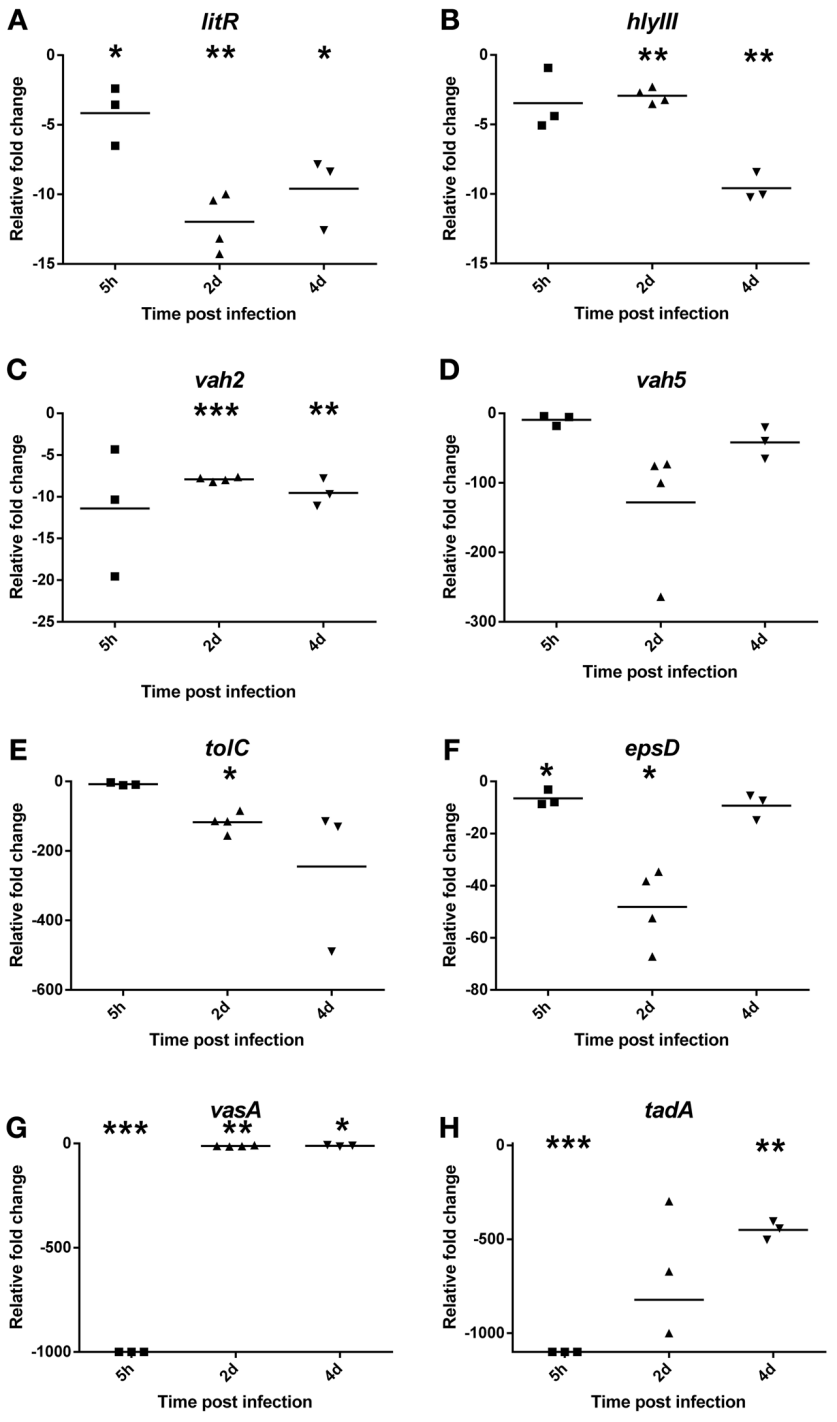
**Relative fold change values of potential bacterial virulence genes (A) *litR*, (B) *vah2*, (C) *vah5*, (D) *hlyIII*, (E) *tolC*, (F) *epsD*, (G) *vasA*, and (H) *tadA* in blood during an experimental *V. salmonicida* i.p.-infection.** Each time point represents the relative expression level of bacterial genes in individual fish and is reported as fold change compared to the average expression levels in bacterial culture (in triplicate) just before challenge. ^*^p < 0.05, ^**^p < 0.005, ^***^p < 0.0005.

To investigate the contribution of bacterial virulence factors to the pathogenesis of *V. salmonicida*, the transcription levels of eight potential virulence genes in blood were evaluated by two-step RT-qPCR (Figure 2). The analysis were performed on samples taken from bacterial culture before challenge (*n* = 2) and from fish 5 h (*n* = 3), 2 days (*n* = 4), and 4 days (*n* = 3) post infection. Average Ct values detected in the bacterial challenge culture were for *litR*: 20.9 ± 0.8, *vah2*: 23.5 ± 0.3, *vah5*: 24.4 ± 0.08, *hlyIII*: 24.9 ± 0.3, *epsD*: 21.2 ± 0.2, *tolC*: 22.1 ± 0.3, *vasA*: 28.2 ± 0.5, and *tadA*: 23.7 ± 0.4.

Compared to transcription values of the *in vitro* bacterial challenge culture, the relative expression of *litR* significantly decreased approximately 4 ± 1.2 (*p* = 0.05), 12 ± 1.1 (*p* = 0.001), and 10 ± 1.5 (*p* = 0.01) fold inside the fish at 5 h and on 2 and 4 days after challenge, respectively (Figure 2A). The relative expression of the three investigated hemolysin genes also decreased after the bacteria were injected into the fish. The change in transcription values from *in vitro* to *in vivo* conditions was only moderate for *vah2* (Mean 4 h p.i.: −9.5 ± 0.96, *p* = 0.003) and *hlyIII* (*x* 4 h p.i. = −9.6 ± 0.57, *p* = 0.0008) showing between 5 and 10-fold decrease for both genes (Figures 2B,C). For *vah5*, however, the relative expression level was more than 100-fold higher *in vitro* compared to 2 days post infection (*x* = −1.8 ± 45.6, *p* = 0.13) (Figure 2D). This result was not statistically significant, however, likely due to high individual variation.

The *in vivo* transcription values of *tolC, epsD*, and *vasA* were also reduced compared to *in vitro*. The relative expression of *tolC* was more than 100-fold higher *in vitro* compared to 2 (−117 ± 14.6, *p* = 0.006) and 4 (−245 ± 122.3, *p* = 0.22) days post infection (Figure 2E). Similar to the transcription profile of *vah5*, transcription values of *epsD* showed a significant 48-fold decrease 2 days after challenge (*x* = −48 ± 7.4, *p* = 0.01) (Figure 2F). No transcription of *vasA* was detected 5 h after challenge (*p* < 0.0001) before the relative expression increased and was observed to be approximately 12-fold lower compared to *in vitro* levels on day 2 (*x* = −12 ± 1.2, *p* = 0.001) and 4 (*x* = −11 ± 1.8, *p* = 0.02) (Figure 2G).

Similar to all the other tested potential virulence genes, *tadA* showed significantly higher transcription rate *in vitro* compared to inside the fish host. Overall, *tadA* was related to largest differences between *in vitro* and *in vivo* gene expression with no transcription products detected 5 h after challenge (*p* < 0.0001), and fold change values of −822 ± 219.5 (*p* = 0.33) and −450 ± 28.6 (*p* = 0.001) on days 2 and 4, respectively (Figure 2H).

### Expression of atlantic salmon immune genes in spleen after challenge with *V. salmonicida*

**Figure 3:**
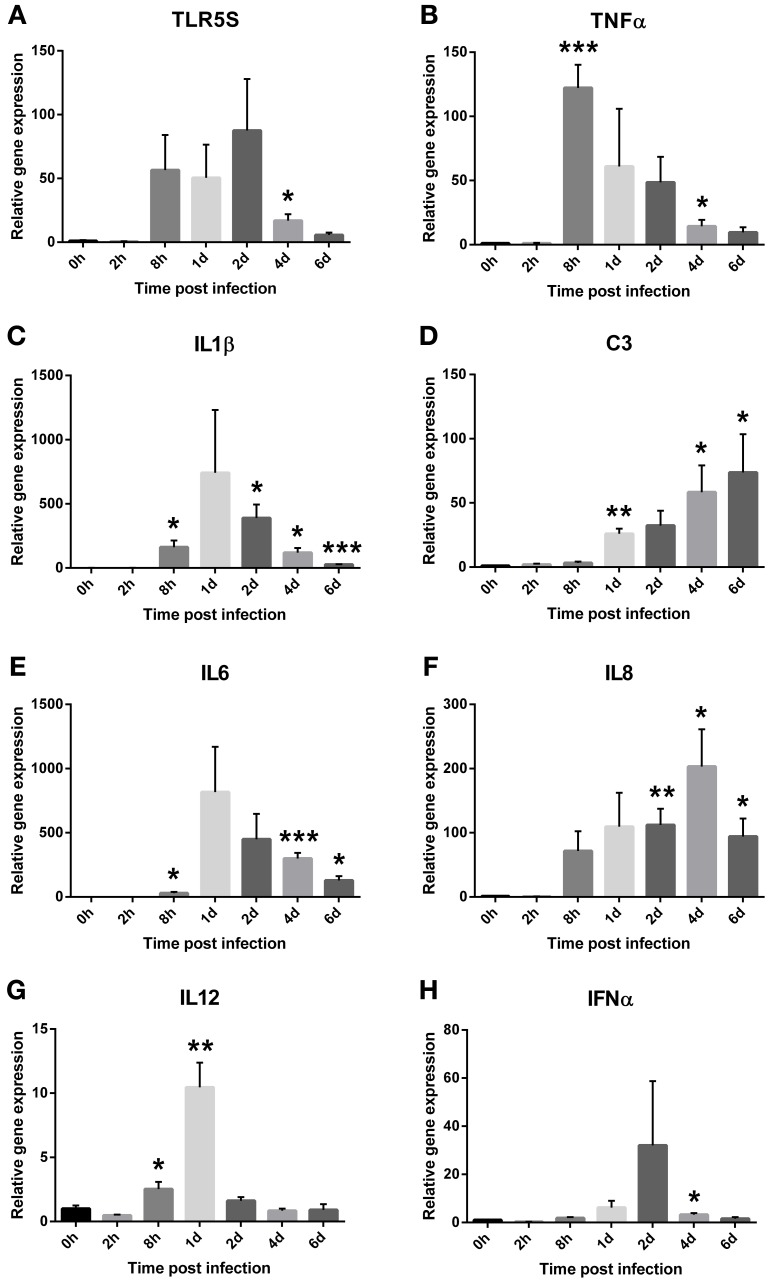
**Relative expression levels of the salmon immune parameters (A) TLR5S, (B) TNFα, (C) IL1β, (D) IL6, (E) IL8, (F) C3, (G) IL12, and (H) IFNα in the spleen during an experimental *V. salmonicida* i.p.-infection.** Each time point represents the average expression level of four fish and is reported as fold change ± standard error of the mean (s.e.m.) compared to the average expression levels of four control fish sampled before challenge. ^*^p < 0.05, ^**^p < 0.005, ^***^p < 0.0005.

The transcription levels of eight immune genes in spleen of Atlantic salmon were evaluated by two-step RT-qPCR after exposure to *V. salmonicida* (Figure 3). Average Ct values detected in control fish before challenge were for TLR5S: 30.9 ± 0.7, TNFα: 29.3 ± 0.4, IL-1β: 30.9 ± 0.7, IL-6: 34.9 ± 0.3, IL-8: 29.0 ± 0.4, IL-12: 29.5 ± 0.2, IFNα: 29.8 ± 0.3, and C3: 31.9 ± 0.8.

Relative gene expression levels of TLR5S remained low in the initial phase of the experiment. The fold changes increased from 8 h post infection (*x* = 56.54 ± 27.51) until day 2, with day 2 showing the overall highest fold change (*x* = 87.55 ± 40.38). From day 4, the relative gene expression decreased with relative fold changes of 16.93 ± 5.04 and 5.69 ± 1.88 on days 4 and 6, respectively (Figure 3A). Significant differences were observed between the challenged and control fish 4 days after challenge (*p* = 0.02). The highest fold change of TNFα was seen 8 h after challenge and was 122-fold higher compared to the control fish (*x* = 122.15 ± 18.03). From this time point, the relative fold changes decreased with time to 60.89 ± 45.06, 48.58 ± 19.88, 14.26 ± 5.02, and 9.57 ± 3.97 on days 1, 2, 4, and 6, respectively (Figure 3B). Significant differences were observed between the challenged and control fish at 8 h (*p* < 0.001) and 4 days (*p* = 0.04) after challenge. The earliest response of IL-1β was observed 8 h after challenge with a fold change of 162 (±51.66, *p* = 0.02). The overall highest relative gene expression level was detected 1 day post infection with 742-fold change higher compared to the control fish (*x* = 742.46 ± 489.99). This result was not statistic significant, however, likely due to high variance. From 2 days post challenge onwards, the relative fold changes decreased significantly with time to 388.75 ± 105.38 (*p* = 0.01), 119.15 ± 36.96 (*p* = 0.02), and 27.16 ± 2.56 (*p* < 0.0001), on days 2, 4, and 6, respectively (Figure 3C).

Relative fold changes of C3 increased with time from low levels 2 and 8 h post infection (2 h *x* = 1.9 ± 0.76, 8 h *x* = 3.21 ± 1.11) to the overall highest level on day 6 (*x* = 73.64 ± 29.92). Significant differences were observed between the challenged and control fish on days 1 (*p* < 0.001), 4 (*p* = 0.03) and 6 (*p* = 0.05) after challenge (Figure 3D). The transcription pattern of IL-6 was similar to IL-1β with the earliest response observed 8 h after challenge (*x* = 29.80 ± 10.15). The highest fold change of IL-6 was the overall highest increase of all immune parameters that were measured and was detected 1 day post infection (*x* = 816.75 ± 353.14). This result was not statistically significant, however, likely due to high variance. From 2 days post challenge, the relative fold changes decreased with time to 448.83 ± 197.55, 299.29 ± 44.27, and 127.91 ± 33.96 on days 2, 4, and 6, respectively (Figure 3E). Significant differences were observed between the challenged and control fish at 8 h (*p* = 0.03), 4 (*p* < 0.001), and 6 days (*p* = 0.01) after challenge. IL-8 transcripts were detected 8 h post infection (*x* = 71.50 ± 30.92) and significantly increased on days 2 (*x* = 112.17 ± 25.33, *p* = 0.005), 4 (*x* = 203.09 ± 58.05, *p* = 0.01), and 6 (*x* = 94.22 ± 27.89, *p* = 0.02) after challenge with the overall highest fold change on day 4 (Figure 3F).

Only small, but significant differences in IL-12 expression were observed in challenged fish compared to the control fish (Figure 3G). At 8 h post infection, transcription levels of IL-12 displayed a 2.5-fold increase (±0.55, *p* = 0.04). The highest level was observed on day 1 and was 10 times (±1.93, *p* = 0.003) higher compared to the control fish. For IFNα, a slight down-regulation was observed 2 h after challenge (*x* = 0.289 ± 0.04) (Figure 3H). After this time point, the relative expression increased to levels above control fish levels, however, similar to the relative expression of IL-12 the fold changes remained low. The highest fold change was observed on day 2 (*x* = 32.01 ± 26.75). Significant differences were observed between the challenged and control fish at 2 h (*p* = 0.0002) and 4 days (*p* = 0.02) after challenge.

## Discussion

### Challenge

To gain more insight into the pathogenesis of CV, an i.p. challenge experiment was performed with *V. salmonicida* in Atlantic salmon. Holding conditions should mimic environmental conditions to limit misinterpretation of the results (Eggset et al., 1997; Johansen et al., 2006). The experiment was therefore performed as closely as possible to a natural CV infection with respect to temperature and host specie. Previous studies have shown that a relative large number of *V. salmonicida* cells are necessary to develop CV in experimentally challenged Atlantic salmon. Bacterial inoculums between 10^5^ and 10^7^ CFU should give a total mortality between 70 and 90% where the lower challenge doses are observed to give a more prolonged disease development compared to the higher doses (Nordmo et al., 1997; Nelson et al., 2007; Bjelland et al., 2012a,b). Injection of a large volume of bacteria to the peritoneal cavity is, however, an artificial way of challenging fish which could affect the result of the study. A previous study has demonstrated that *V. salmonicida* rapidly invades the host's blood stream after bath as well as i.p. challenge (Bjelland et al., 2012a). Based on these findings, it was assumed that the expression of bacterial virulence and host immune genes in the fish blood is irrespective to challenge model and comparable with a natural infection of *V. salmonicida*.

### Evaluation of potential reference genes in qPCR studies

To date, no studies have been performed to identify suitable candidate genes of *V. salmonicida* for normalization in qPCR based gene expression analysis. The present study therefore evaluated the suitability of six housekeeping genes as potential reference genes for expression *in vitro* as well as in blood and spleen tissue. The housekeeping genes *accD, gapA*, and *16S rDNA* were found to be the most stable expressed reference genes and further used for normalization of gene expression in *V. salmonicida*. A previous study has evaluated the stability of potential reference genes in qPCR studies of Atlantic salmon and ranked the stability of examined housekeeping genes in spleen as *EFN1β* > *S20* > *18S rDNA* > *β-actin > EFN1α > GAPDH* (Olsvik et al., 2005). Initially, the three most stable genes *EFN1β, S20*, and *18S rDNA* were chosen as reference genes in our study. However, the *S20* primer pair showed a double peak in the dissociation curve and subsequent attempts to optimize the reaction conditions did not solve the problem. Thus, *β-actin* was included in the reference gene panel. In contrast to the study of Olsvik et al. (2005), the most stable housekeeping genes in spleen were in our study ranked as *β-actin* > *EFN1β* > *18S rDNA*. The differences observed between the two studies could be related to factors as fish size, numbers, origin and health status since the study of Olsvik et al. included tissues from 15 healthy individuals with a weight range from 254 to 1898 g, while the present study included tissues from 28 fish, both healthy and infected, of approximately the same weight (50 g). Nevertheless, this demonstrates the importance of a careful selection of appropriate housekeeping genes for accurate and reliable normalization of gene expression data.

### Quantification of *V. salmonicida* growth in tissue samples using two-step RT-qPCR

The ability of *V. salmonicida* to rapidly establish a bacteremia has previously been demonstrated. The time span from the establishment of bacteremia until symptoms of CV are registered implies a latency period. It is suggested that this latency period is needed for bacterial proliferation to overcome the host's immune defense (Bjelland et al., 2012a). Although a large number of bacteria are reported in internal organs of moribund fish, no previous studies have been performed to evaluate the growth of *V. salmonicida in vivo* (Egidius et al., 1986; Totland et al., 1987). Thus, the relative expression of the stable bacterial reference gene *16S rDNA* was used to illustrate the bacterial growth in the fish blood during challenge. Semi-quantification of bacteria from the blood and head kidney by traditional culturing supported the two-step RT-qPCR results. This is in accordance with previous studies and demonstrates that traditional methods produce reliable results with high time- and cost-effectiveness (Løvoll et al., 2009; Bjelland et al., 2012a). The bacterial concentration remained low during the first 2 days of experiment. From day 4, a significant increase in bacterial transcripts was observed and the overall highest concentration of *V. salmonicida* was detected on day 6 when the experiment was terminated. These results are in accordance with the previous hypothesis that *V. salmonicida* requires a latency period in the blood stream for sufficient bacterial proliferation. The results illustrate a typical exponential bacterial growth curve starting with a prolonged lag phase where the bacterium adapts to the growth conditions and the host immune response. The latency period is followed by an exponential growth phase characterized by cell doubling at regular intervals. During this period, the bacterial growth is not limited by nutrition and host defense molecule, thus the host seems to be overwhelmed by the bacterial intruder. The present study was terminated on day 6 when the bacterial population still was in the exponential growth phase indicating a generalized septicemia.

### Expression of potential *V. salmonicida* virulence genes during a CV infection

To compare the expression of potential virulence factors during *in vitro* and *in vivo* conditions and thus further elucidate the pathogenesis of *V. salmonicida*, the transcription levels of eight potential virulence genes were evaluated by two-step RT-qPCR. Surprisingly, the potential virulence genes showed in general highest transcription levels *in vitro* compared to inside the fish host.

It has previously been demonstrated that the quorum sensing master regulator LitR negatively regulates adhesion, aggregation and biofilm production in addition to impacting virulence in *V. salmonicida*. Thus, *V. salmonicida* LitR is suggested to be an important factor for adapting the bacterium from a sea water living “biofilm mode” to a “planktonic mode” more suitable for infection (Bjelland et al., 2012b). Our study aimed to further investigate these hypotheses by *in vivo* expression analysis of *litR*. During an infection in a nutrition rich environment such as the fish tissue, increasing cell densities of *V. salmonicida* and expression of LitR should down-regulate this “biofilm mode.” Surprisingly and in contrast to the previous *in vitro* study, the *litR* expression per cell *in vivo* was found to be cell-density independent. However, the increasing numbers of LitR-expressing *V. salmonicida* cells throughout the trial could likely maintain the planktonic mode.

It has been hypothesized that bacterial extracellular toxins are responsible for the extended petechial hemorrhages observed during a CV infection (Holm et al., 1985; Totland et al., 1987). With a few exceptions (on blood from mice and rabbit), production of hemolysins *in vitro* has never been described in *V. salmonicida*. However, genomic analysis have identified three putative hemolysins *vah2, vah5*, and *hlyIII* that shows 77, 52, and 70% identity to hemolysins of *V. anguillarum (vah2* and *vah5)* and *V. vulnificus (hlyIII)*, respectively (Hjerde et al., 2008). The hemolytic activity of the *V. anguillarum vah* genes (*vah1–5*) has been considered to be the virulence factor responsible for hemorrhagic septicemia during vibriosis (Hirono et al., 1996; Rodkhum et al., 2005). Similarly, the *hlyIII* hemolysin of *V. vulnificus* is also demonstrated to play a role in virulence (Chen et al., 2004). By taking these previous reports into consideration, it was expected to observe an increase in production of hemolysins in this study. Most surprisingly the results came out to be the complete opposite. The results indicate that the expression of *vah2* and *hlyIII* is only moderately changed after entering the fish host. Thus, any significance to the pathogenesis of CV of this down-regulation is highly speculative. For *vah5*, however, the decrease in gene expression *in vivo* was significant and this result may indicate that the Vah5 protein in some way plays a role outside the fish host. The extensive hemolysis occurring in CV may then be a result of the activity of the immune system of the salmon.

Different types of secretion systems are described among *Vibrio* species and some of these are showed to be related to virulence e.g., rtx toxin and cholera toxin are secreted through type I and type II secretion systems, respectively. In *V. salmonicida*, the genes for six different secretion systems are identified and includes three type I (T1SSI, T1SSII, and T1SSIII), one type II (T2SS) and two type VI (T6SSI and T6SSII) secretion systems (Hjerde et al., 2008). Thus, the presence of these systems in *V. salmonicida* demonstrates that the bacterium has the tools required for the secretion of extracellular toxins and/or enzymes. To investigate the impact of secretion systems inside the fish host, our study included gene expression analysis of the three genes *tolC, epsD*, and *vasA* that code for essential proteins of *V. salmonicida* T1SSII, T2SS, and T6SSI, respectively. Equal to the other potential virulence genes investigated, *tolC, epsD*, and *vasA* showed highest transcription levels *in vitro* compared to inside the fish host. The similar transcription profiles of *epsD* and *vah5* might suggest that the hemolysin is secreted through T2SS. This hypothesis is, however, highly speculative and requires further investigations. Only three of six secretion systems identified in *V. salmonicida* were included in this study. Hence, there is a possibility that the remaining three systems are expressed in different ways compared to T1SSII, T2SS, and T6SSI. Nevertheless, the unanimous down-regulation of the investigated secretion system genes suggests that extracellular toxins and enzymes is of minor importance during the CV disease development.

Adhesion of bacteria to the host surface is one of the initial steps in microbial pathogenesis (Taylor, 1991; Kline et al., 2009). In *V. salmonicida*, genomic analysis has identified coding sequences for a flp-type pilus system. Flp-type pilus system is described in several bacteria to impact auto-aggregation and unspecific adherence, and in some species e.g., *Actinobacillus pleuropneumoniae* the system also impacts virulence (Li et al., 2012). In contrast, the flp-type pilus system of the fish pathogen *Aeromonas salmonicida* subsp. *salmonicida* is shown to make little or no contribution to the development of furunculosis (Boyd et al., 2008). Similar to the expression of the *V. salmonicida* T6SS *vasA* gene, *tadA* expression was totally absent 5 h after challenge. The muting of *tadA* after entering the fish host is in accordance with the previous presumption that down-regulation of adhesion properties is mandatory for adapting *V. salmonicida* to become a virulent fish pathogen. Thus, these results indicate that *tadA* mainly plays a role in bacterial survival during environmental stages of life. T6SS is reported to be related to highly variable phenotypes among different bacteria including biofilm formation (Aschtgen et al., 2010; Records, 2011; Liu et al., 2012). Thus, similar to *tadA*, the complete down-regulation of *vasA* could also be related to bacterial adaptation from the environment to the fish host.

The virulence gene expression study elicited the question about why *V. salmonicida* down-regulate many important and potential virulence properties after infecting the host. Obviously, the genes analyzed in this study may not be important virulence factors in *V. salmonicida* and more potential virulence genes should be included in future studies. qPCR is a precise and reproducible technology for assaying the expression of a small number of genes. However, with a large number of samples to test against each target gene, the qPCR technology has limitations when it comes to time and cost effectiveness. To identify large numbers of important pathogen and host genes methods such as micro-arrays and transcriptomics could be better choices (Moreira et al., 2012; Rader and Nyholm, 2012; Montanchez et al., 2013). The RT-qPCR method, however, is demonstrated to have a greater dynamic range and therefore allow for a more precise quantitation of expression levels than the high-throughput methods (Dallas et al., 2005). Nevertheless, these results might indicate that a general muting of gene expression is the bacterium's strategy to hide from the host immune defense system.

### Expression of atlantic salmon immune genes during a CV infection

To date, no studies have been conducted to investigate the innate immune response in Atlantic salmon during a CV infection. Therefore, to evaluate the results from the immune parameter analysis, previous reports from bacterial challenge experiment on salmonid fish were used for comparison (Løvoll et al., 2009; Raida and Buchmann, 2009; Ching et al., 2010; Hynes et al., 2011; Zhang et al., 2011; Aykanat et al., 2012; Kvamme et al., 2013). Toll-like receptor 5 (TLR5) plays an essential role in the innate immune defense against bacterial invasion through the recognition of the bacterial protein flagellin followed by the activation of various proinflammatory cytokines (Takeda et al., 2003). In salmonids, TLR5 exists both in a membrane bound (TLR5M) and a soluble (TLR5S) form. Hynes et al. (2011) have reported a significantly increase of TLR5S on day 4 with a fold change of 20–35 when Atlantic salmon was injected with purified flagellin. Another study that performed cohabitant challenge of Atlantic salmon with *Aeromonas salmonicida* has shown an approximately 2.5 and 10-fold increase of TLR5S after 3 and 14 days, respectively (Zhang et al., 2011). Furthermore, Raida and Buchmann (2009) have challenged rainbow trout fry with *Yersinia ruckeri* i.p. and showed that the expression of TLR5S in liver was 3.6-fold higher after 8 h followed by a steady decrease down to control fish expression levels.

The recognition of pathogen associated molecules such as LPS and flagella result in a proinflammatory cytokine cascade whereby TNFα is released followed by IL-1β and then IL-6 (Secombes et al., 2001). In two separate vaccine experiments using *V. anguillarum* antigens, the transcript levels of TNF1α was detected to be low (4-fold increases) or absent (Aykanat et al., 2012; Kvamme et al., 2013). In contrast, experiments with live *Vibrio anguillarum* demonstrated an approximately 15, 25, and 5-fold increases of TNFα expression 12, 24, and 72 h after i.p. challenge, respectively (Ching et al., 2010). More elevated levels of IL-1β have also been demonstrated after challenge with live bacteria than seen after vaccination. Ching et al. have reported an increasing expression of IL-1β from 20-fold changes after 6 h to approximately 100-fold changes 3 days after i.p.-challenge. In similar, an immersion challenge with *Moritella viscosa* in Atlantic salmon reported the first response of IL-1β after 2 days. The transcription level increased during the next days with the peak expression on day 7 (50–220-fold increase) (Løvoll et al., 2009). For IL-6, Hynes et al. (2011) have reported that the highest increase in expression was seen on day 4 (fold change of 78) followed by a significantly decrease. A similar profile has been demonstrated by Raida and Buchmann (2009) where IL-6 showed a significantly increase in fold changes at 8 h (4-fold), on days 1 (46-fold) and 3 (1100-fold) post infection, followed by a decrease in transcription levels.

In the present study, a minority of the results from the immune parameter experiment were statistically significant most likely due to variation between the fish's individual immune response (Lohm et al., 2002). To increase the probability for obtaining more significant results, more fish could have been included in the trial. However, the number of experimental animals is a balance between uncertainty in the expected results and the need to keep the number of animals at a low level. Another methodological consideration is that although the overall pattern of protein expression is similar to that of mRNA expression, the correlation between mRNA and protein levels may vary (Tian et al., 2004; Shebl et al., 2010). This could be explained by posttranscriptional and posttranslational regulation and by misclassification due to measurement errors including the total RNA content of the sample, the number of cells in the starting material, the RNA extraction efficiency and differential enzymatic efficiencies (Vandesompele et al., 2002). Thus, the use of proteomic techniques might help improve our understanding of the relationship between mRNA expression and protein production.

Nevertheless, the pattern of gene activation and expression suggests that the innate response during cold-water vibriosis is based on the pathogen's ability to engage TLRs signaling pathways to trigger the modulation of cytokines, in addition to a subsequent production of the complement component 3 (C3). *V. salmonicida* seems to induce a rapid and strong response of TLR5S in Atlantic salmon, with a relative fold change of more than 50 from 8 h to 2 days after challenge. However, as the bacterial growth became exponential after day 2, the transcription level of TLR5S decreased to lower levels. The gene expression of the three cytokines TNFα, IL-1β, and IL-6 showed similar profiles. The highest fold changes were observed after 8 h (TNFα) and 1 day (IL-1β and IL-6) and were 122, 742, and 817, respectively. These high peaks were all followed by a step-by-step decrease to low levels on day 6. It should be noted that the former studies used to compare our results include different fish species, infective agents and challenge methods. Nevertheless, our results demonstrate that *V. salmonicida* is recognized by the host and induces a rapid and strong, but short-lasting immune response. This indicates that *V. salmonicida* is resistant against important salmon cytokines and may also possess mechanisms that inhibit the host immune response during a CV infection.

Downstream of TNFα, IL-1β, and IL-6 other cytokines are released such as IL-8 that induce migration of neutrophils and phagocytes to the site of infection (Secombes et al., 2001; Zhu et al., 2013). Two previous studies have reported a rapid and small to moderate (5–15-folds) increase in IL-8 gene expression during challenge (Ching et al., 2010; Aykanat et al., 2012). In contrast, a later induction of IL-8 has also been reported (Hynes et al., 2011). In the present study, transcription products of IL-8 were identified 8 h after infection. However, in contrast to the other early detected cytokines TNFα, IL-1β, and IL-6, the gene expression of IL-8 demonstrated a more stable profile with fold values about 100 throughout the experiment. This chemokine attracts immune cells to the site of infection and our results may suggest that the increasing amount of bacteria during the infection maintain the strong stimulation of IL-8.

The cleavage of C3 gives e.g., the opsonin C3b that binds microbial cells and enhances phagocytosis (Nakao et al., 2011). The gene expression of C3 in Atlantic salmon has by Løvoll et al. (2009) been described to be induced 2 days after bath challenge with *M. viscosa* and followed by an increase throughout the study with a peak expression of 100–250-fold changes on day 7. Our study demonstrates similar results with increasing C3 gene expression levels from approximately three-fold changes 8 h after challenge to 74-fold changes on day 6. These results demonstrate that *V. salmonicida* has developed resistant mechanisms against the bactericidal effect of complement. Previously, it has been hypothesized that the host initiate a strong inflammatory response against *V. salmonicida* that eventually damage its own cells and tissue. The complement system has the potential to be extremely damaging to self-tissues, meaning its activation must be tightly regulated. Perhaps C3 is a factor that could address the pathological signs observed during CV.

The binding of microbial antigens to host recognition receptors also stimulate the transcription and secretion of the cytokines IL-12 and IFNα. IL-12 is the predominant cytokine driving the differentiation of naive T helper cells into TH1 cells (Ho and Glimcher, 2002). IFNα is mainly involved in antiviral defense (Robertsen, 2006). Similar to the report of Hynes et al. (2011), the present gene expression results of IL-12 and IFNα was mostly low or absent. IFNα was used as a marker for intracellular bacterial growth. Thus, the low or absent expression of IFNα indicates that the *V. salmonicida* pathogenesis mostly takes place extracellular. The low expression of IL-12 could inhibit the differentiation of T helper cells followed by a negative effect on the production of cytokines.

## Concluding remarks

In this work we have demonstrated that *V. salmonicida* proliferates in the fish blood after an initial latency period. To do this, *V. salmonicida* must have developed a proper strategy to resist the bactericidal effect of serum and avoid the immune cells of the blood stream.

It has earlier been suggested that the bacterium is hiding from the host immune system during the latency period and in this way increase in population size to ensure a successful infection. The muting of the potential virulence genes *in vivo* supports this hypothesis. In contrast, the immune parameter results demonstrate an initial rapid and strong immune response indicating that the pathogen is recognized by the salmon host. The immune response against *V. salmonicida* seems, however, to be short-lasting. This indicates that the bacterium possesses mechanisms that inhibits the development of a proper defense against CV or just simply uses resistance mechanisms to avoid the fish immune system. Finally, the host will become completely exhausted of fighting the overwhelming infection.

### Conflict of interest statement

The authors declare that the research was conducted in the absence of any commercial or financial relationships that could be construed as a potential conflict of interest.
